# Phenolic Profile of Grape Canes: Novel Compounds Identified by LC-ESI-LTQ-Orbitrap-MS

**DOI:** 10.3390/molecules24203763

**Published:** 2019-10-18

**Authors:** Danilo Escobar-Avello, Julián Lozano-Castellón, Claudia Mardones, Andy J. Pérez, Vania Saéz, Sebastián Riquelme, Dietrich von Baer, Anna Vallverdú-Queralt

**Affiliations:** 1 Department of Nutrition, Food Science and Gastronomy XaRTA, Institute of Nutrition and Food Safety (INSA-UB), Faculty of Pharmacy and Food Sciences, University of Barcelona, 08028 Barcelona, Spain; daniescobar01@gmail.com (D.E.-A.); julian.lozano@ub.edu (J.L.-C.); 2 Unidad de Desarrollo Tecnológico, Universidad de Concepción, 4191996 Coronel, Chile; sebastianriquelmerifo@gmail.com; 3 Consorcio CIBER, M.P. Fisiopatología de la Obesidad y la Nutrición (CIBERObn), Instituto de Salud Carlos III (ISCIII), 28029 Madrid, Spain; 4 Departamento de Análisis Instrumental, Facultad de Farmacia, Universidad de Concepción, Concepción, Chile; cmardone@udec.cl (C.M.); aperezd@udec.cl (A.J.P.); vaniastepsaezpulg@gmail.com (V.S.); dvonbaer@gmail.com (D.v.B.)

**Keywords:** grape canes, polyphenols, LC–LTQ-Orbitrap, *Vitis vinifera*, by-products

## Abstract

Grape canes (*Vitis vinifera* L.) are a viticulture industry by-product with an important content of secondary metabolites, mainly polyphenols with a broad spectrum of demonstrated health benefits. Grape canes, therefore, have considerable economic potential as a source of high-value phytochemicals. In this work, liquid chromatography coupled with electrospray ionization hybrid linear trap quadrupole-Orbitrap mass spectrometry (LC–LTQ-Orbitrap) was used for the comprehensive identification of polyphenolic compounds in grape canes. Identification of polyphenols was performed by comparing their retention times, accurate mass measured, and mass fragmentation patterns with those of reference substances or available data in the literature. A total of 75 compounds were identified, including phenolic acids, flavanols, flavonols, flavanonols, flavanones, and stilbenoids. The most abundant polyphenols were proanthocyanidins and stilbenoids and their oligomers. Moreover, the high-resolution mass spectrometry analysis revealed the occurrence of 17 polyphenols never described before in grape canes, thereby providing a more complete polyphenolic profile of this potentially valuable by-product.

## 1. Introduction

The bark of woody plants considered a by-product of the forestry agricultural, and wood industry. This can be an abundant source of polyphenolics compounds with high recovery yields [1,2,3]. Bark polyphenols might be esterified and used for the design of thermoplastic blends [4,5] and the developing of adhesive resins [6]. However, the most extended application of bark polyphenols compounds is its biological effects, such as antioxidant, anti-inflammatory, anti-tumor, antidiabetic, antimutagen, etc. [1,7]. In particular, grape canes, known also as vine-shoots, are one of the most important by-products in viticulture, alongside grape seeds, pomace, stalks, and skins, all of which could provide low-cost raw material for the production of high-value phytochemicals because of their rich polyphenolic content.

Polyphenolic compounds, the most important class of secondary metabolites in *V. vinifera* L., are synthesized by the phenylpropanoid pathway in response to biotic and abiotic stimuli [8]. The most abundant polyphenols in grape canes are oligostilbenoids and proanthocyanidins [9,10]. Stilbenoids are members of the non-flavonoid phenolic family and play an important role in the defense mechanism of plants. The concentration of stilbenoids in grape canes strongly depends on their storage/treatment after pruning [11]. Proanthocyanidins are oligomers and polymers formed by flavan-3-ol units with multiple possible linkages and different degrees of polymerization [10].

Stilbenoids, mainly (*E*)-resveratrol, have a wide range of health benefits, with positive effects on cardiovascular and cognitive diseases, cancer, type 2 diabetes, oxidative stress, and inflammation states [12]. Proanthocyanidins exhibit a broad spectrum of pharmacological and therapeutic benefits, including prevention of oxidative stress and degenerative diseases, gastrointestinal distress, neurological disorders, pancreatitis and various stages of neoplastic processes and carcinogenesis [13]. Besides stilbenoids and proanthocyanidins, grape canes contain other polyphenolic compounds with high biological value but in lower concentrations. The unique combination of grape phenolic compounds makes grape, raisins and grape canes, a promising source for the development of novel nutraceutical products [14].

Although various polyphenols have been identified and quantified in grape canes [9,10,11,15,16,17,18,19,20,21,22,23], a comprehensive chemical profiling is still needed, particularly of specific identities for proanthocyanidins and some polyphenolic derivatives. For the structural elucidation of unknown compounds in complex samples, the high-resolution/accurate mass system, LTQ-Orbitrap-MS, has proven to be a reliable tool [24,25,26,27,28,29,30].

The aim of the present work was to provide an accurate and comprehensive identification of polyphenols in grape canes using liquid chromatography coupled with electrospray ionization hybrid linear trap quadrupole-Orbitrap mass spectrometry (LC–LTQ-Orbitrap) analysis, with special focus on previously unreported compounds. The novelty of this study is to extend the knowledge about polyphenols identity of grape canes for the development of additives in food, cosmetics, biomaterials, and other biobased products.

## 2. Results and Discussion

### 2.1. General

**Figure 1:**
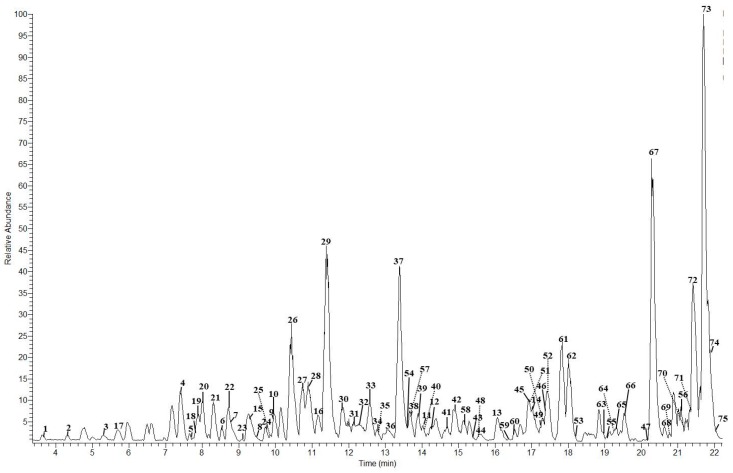
Base peak chromatogram of grape cane. Peaks and compounds are shown in Table 1.

Table 1 shows the 75 polyphenolic compounds identified in grape canes through LC–LTQ-Orbitrap experiments, along with their retention times (min), accurate mass, ion molecular formula (IMF), error (ppm), and the MS^2^ ions used for identification. The main polyphenolic classes identified were: hydroxybenzoic acids (**14**), hydroxycinnamic acids (**2**), flavanols (mainly proanthocyanidins) (**31**), flavonols (**3**), flavanonols (**3**), flavanones (**3**), and stilbenoids (**19**). To the best of our knowledge, 17 polyphenols were identified for the first time in grape canes, although some of them have been previously identified in other wine by-products, such as grape seeds [31,32,33], stalks [34], pomace [27,35] and skins [31,33,36]. Figure 1 shows a base peak chromatogram of a grape cane extract.

### 2.2. Phenolic Acids

Phenolic acids, abundant in agro-industrial by-products [37], are of interest for their biological activity as anti-inflammatory, hepatoprotective, antioxidant, antimicrobial, cardioprotective, antidiabetic, anticancer, and neuroprotective agents [38]. Phenolic acids identified in grape cane extract can be subdivided into hydroxybenzoic and hydroxycinnamic acids and their derivatives.

#### 2.2.1. Hydroxybenzoic Acids and Derivatives

A total of fourteen hydroxybenzoic acids were identified in the grape cane extract (Table 1). The typical neutral loss of CO_2_ (−44 Da) was observed for: gallic acid (*m*/*z* 169.0141, peak 2), protocatechuic acid (*m*/*z* 153.0192, peak 5), 2-hydroxybenzoic acid (*m*/*z* 137.0243, peak 8), 4-hydroxybenzoic acid (*m*/*z* 137.0242, peak 10), and ellagic acid (*m*/*z* 300.9980, peak 14). Moreover, gallic, 4-hydroxybenzoic and ellagic acids were confirmed by comparing the retention time and MS^2^ spectra with available standards. The presence of these compounds, with the exception of 2-hydroxybenzoic acid (*m*/*z* 137.0243, peak 8), was also recently reported in Portuguese vine shoot wastes [39].

Seven hexoside derivatives of hydroxybenzoic acids were detected in the grape cane extract: monogalloyl-glucose (*m*/*z* 331.0668, peak 1; *m*/*z* 331.0664, peak 3), protocatechuic acid-*O*-hexoside (*m*/*z* 315.0719, peak 4; *m*/*z* 315.0718, peak 6), syringic acid hexoside (*m*/*z* 359.0981, peak 7), hydroxybenzoyl hexoside (*m*/*z* 299.0770, peak 9), and ellagic acid hexoside (*m*/*z* 463.0518, peak 11). The MS^2^ spectra showed the characteristic fragmentation involving cleavage of the hexosyl moiety (−162 Da) [25]. Additionally, both monogalloyl-glucoses showed product ions at *m*/*z* 271 and 211, probably generated by the fragmentation of the hexose moiety (−60 Da) [26] and removal of two formaldehyde (CH_2_O) groups in the glucose moiety, respectively [40]. Previous studies have identified and quantified phenolic acids (e.g., gallic, protocatechuic, syringic and ellagic acids) in vine shoot extracts [39], but to the best of our knowledge, this is the first report of hydroxybenzoic acid hexoside derivatives in grape canes.

Interestingly, two hydroxybenzoic acid derivatives were also identified. Gallic acid ethyl ester (*m*/*z* 197.0454, peak 12) showed an ion at *m*/*z* 169 arising from the loss of an ethyl unit (−28 Da). Gallic acid ethyl ester or ethyl gallate have been previously identified in wine extracts [41], but not in grape canes. Ellagic acid pentoside (*m*/*z* 433.0416, peak 13) was also identified and confirmed by MS^2^ experiments. In the MS^2^ spectrum of *m*/*z* 433, the ion at *m*/*z* 301 was due to the loss of a pentosyl unit (−132 Da) [42]. As far as we know, this is the first time that ellagic acid pentoside has been identified in grape canes.

#### 2.2.2. Hydroxycinnamic Acids Derivatives

Hydroxycinnamic acids are important polyphenol precursors biosynthesized in plants from the amino acids phenylalanine and tyrosine in the shikimate pathway [43]. Hydroxycinnamic acids and their derivatives exhibit antioxidant, anti-inflammatory, antimicrobial, and ultraviolet protective effects, suggesting a potential application in anti-aging and anti-inflammatory products [44].

Two hydroxycinnamic acids were identified: (i) caftaric acid (*m*/*z* 311.0406, peak 15), with ions at *m*/*z* 179 (caffeic acid) and 149 (tartaric acid) due to the loss of a tartaric acid moiety (−132 Da) and the presence of a tartaric acid molecule, respectively [45]; and (ii) coutaric acid (*m*/*z* 295.0456, peak 16), with an ion at *m*/*z* 163 attributed to a coumaric acid molecule observed after the loss of tartaric acid (−132 Da) [31,45]. Caftaric and coutaric acids have been previously identified and quantified in wine and vine shoot extracts [22,23].

### 2.3. Flavanols (Proanthocyanidins)

**Figure 2:**
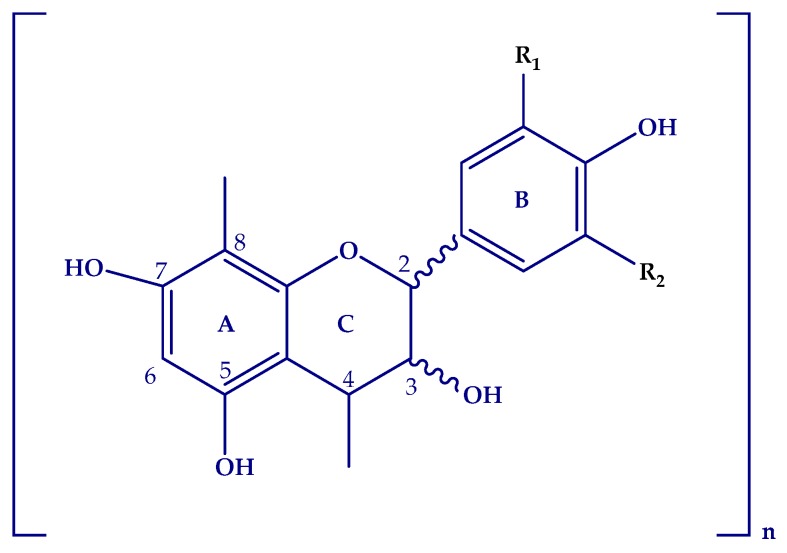
Flavonoid skeleton. Procyanidins: R_1_=H, R_2_=OH; Prodelphinidins R_1_=OH, R_2_=OH. Adapted from [46].

Proanthocyanidins identified in grape cane extracts can be subdivided into procyanidins with a 3′,4′-dihydroxy substitution and prodelphinidins with a 3′,4′,5′-trihydroxy substitution, both on the B ring (Figure 2).

#### 2.3.1. Procyanidins and Monomers

The flavan-3-ols (+) catechin (*m*/*z* 289.0715, peak 29) and (−) epicatechin (*m*/*z* 289.0715, peak 37) were confirmed after comparison with standards. In the MS^2^ spectrum of *m*/*z* 289, ions at *m*/*z* 245 [M − H − 44]^−^ could be attributed to the loss of –CH_2_–CHOH– or CO_2_ moieties [47,48] and at *m*/*z* 205 and 179 to the loss of the flavonoid A ring [M − H − 84]^−^ and B ring [M − H − 110]^−^ [48], respectively. (+) Catechin and (−) epicatechin have been widely reported and quantified in grape canes [17,23,39].

Several procyanidins with different degrees of polymerization (DP) were found. Procyanidins show various fragmentation pathways derived from quinone methide (QM), heterolytic ring fission (HRF), and retro-Diels-Alder (RDA) mechanisms [49].

Five procyanidin dimers (DP2) (*m*/*z* 577.1342, peak 26; *m*/*z* 577.1347, peak 28; *m*/*z* 577.1348, peak 32; *m*/*z* 577.1356, peak 33; *m*/*z* 577.1346, peak 40) were detected. Procyanidins B1, B2, and C1 were confirmed by MS^2^ and standard in previous works [9,17]. The MS^2^ spectrum of *m*/*z* 577 produced ions at *m*/*z* 451, 425, 407 and 289. The *m*/*z* 451 was attributed to HRF of the C ring with a characteristic loss of 126 Da. The ion at *m*/*z* 425 was due to RDA fragmentation with a neutral loss of 152 Da, followed by the loss of a water molecule unit (−18 Da) at *m*/*z* 407 [M − H − 152 − 18]^−^. The ion at *m*/*z* 289 was due to QM fission of the interflavan bond producing a distinctive loss of 288 Da [35].

Four procyanidin trimers (DP3) (*m*/*z* 865.1982, peak 19; *m*/*z* 865.1978, peak 30; *m*/*z* 865.1979, peak 41; *m*/*z* 865.1959, peak 43) were tentatively identified. In previous studies, two procyanidin trimers, including procyanidin C1, were identified using a QTrap 3200 LC/MS/MS system [17]. The higher sensitivity of the LTQ-Orbitrap system used in the current study allowed the identification of the other two procyanidin trimers. MS^2^ of *m*/*z* 865 produced peaks at *m*/*z* 739, 713, 695, 577, 451, 425, 407, and 289. The ion at *m*/*z* 739 was due to HRF [M − H − 126]^−^ of the C ring (of the upper unit). The ions at *m*/*z* 713 and *m*/*z* 695 were produced by the RDA mechanism [M − H − 152]^−^, followed by the loss of a water molecule [M − H − 152 − 18]^−^. The ion at *m*/*z* 577 was generated by a QM [M − H − 288]^−^ cleavage between the C and D rings. The remaining ions at *m*/*z* 451, 425, 407, and 289 could be explained as for the procyanidin dimers above.

One procyanidin tetramer (DP4) (*m*/*z* 576.1271 [M − 2H]^2−^, peak 31) with doubly charged ions was detected, whose fragmentation gave rise to ions at *m*/*z* 1027, 865, 863, 739, 451, 407, 289 and 287. The ion at *m*/*z* 1027 was produced by HRF [M − H − 126]^−^ of the tetrameric unit [50]. The ions at *m*/*z* 865 and 863 arose from QM [M − H − 288]^−^ cleavage of the interflavan bond in the top and second units. Another ion at *m*/*z* 287 was generated by QM fission due to the ion at *m*/*z* 863 [M − H − 288 − 288]^−^. Tetrameric procyanidins have been previously detected in grape canes [19]. The ions at *m*/*z* 739, 451, 407, and 289 are explained above.

**Figure 3:**
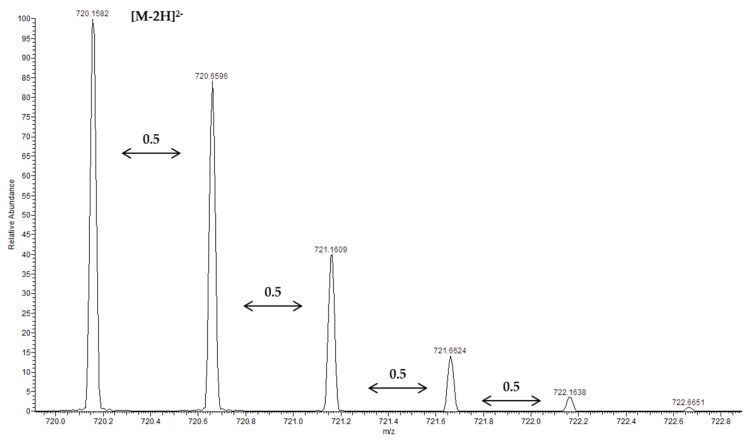
Mass spectra showing the doubly charged ions of the procyanidin pentamer.

Two procyanidin pentamers (*m*/*z* 720.1580 [M − 2H]^2−^, peak 36; *m*/*z* 720.1578 [M − 2H]^2−^, peak 44) were also detected as doubly charged ions, as confirmed by the mass difference of 0.5 Da between the isotopic peaks (Figure 3). Fragmentation of both doubly charged ions gave ions at *m*/*z* 1315, 1153, 1151, 1027, 865, 863, 739, 635, 577, 575, 451, 407, 289, and 287. The ion at *m*/*z* 1315 was produced by the HRF [M − H − 126]^−^ fragmentation pathway from the ion at *m*/*z* 1441. The ions at *m*/*z* 1153 and 1151 were derived from a QM-type cleavage. The ion at *m*/*z* 635 has been previously observed in a procyanidin pentamer in grape stalk extracts [34]. The product ions at *m*/*z* 1027, 865, 863, 739, 577, 575, 451, 407, 289, and 287 are explained above for other oligomeric procyanidins. As far as we know, this is the first time that procyanidin pentamers have been identified in grape canes.

#### 2.3.2. Prodelphinidins and Gallate Derivatives

Prodelphinidins have been previously identified in grape canes using a two-dimensional liquid chromatography-based method [10], but the use of a high-resolution mass analyzer, such as the LTQ Orbitrap MS, could be used by improves their characterization.

Epicatechin gallate (*m*/*z* 441.0825, peak 45) was confirmed by comparison with the standard. The MS^2^ spectrum of epicatechin gallate produced two fragment ions arising from the cleavage of the ester bond: at *m*/*z* 289 for deprotonated epicatechin and at *m*/*z* 169 for a deprotonated gallic acid moiety [25]. Epicatechin gallate has been previously identified in vine shoots of the Airén and Cencibel varieties [22].

Three monogallate procyanidin dimers (*m*/*z* 729.1459, peak 39; *m*/*z* 729.1449, peak 42; *m*/*z* 729.1441, peak 46) were also identified. The peaks 39 and 46 were tentatively assigned as (epi)catechin gallate (ECG)→(epi)catechin, and produced ions at *m*/*z* 603, 577, 439, 425, 407, and 289. The ion at *m*/*z* 603 corresponded to the loss of the A ring (1,3,5-trihydroxybenzene) (−126 Da) of the upper elemental unit via RDA fission [51]. The ions at *m*/*z* 577, 425, 407 and 289 showed the same fragmentation pattern as described for procyanidin dimers. The ion at *m*/*z* 439 was due to QM fission and was crucial in assigning the gallic acid ester in the upper position [51,52]. In addition, the compound at peak 42 was tentatively identified as (epi)catechin→(epi)catechin gallate, with ions at *m*/*z* 603, 577, 451, 441, 407, and 289. The main difference with peaks 39 and 46 was the presence of an ion at *m*/*z* 441 due to QM cleavage. This fragment unambiguously confirms that the gallic acid ester is at the bottom position [51]. Procyanidin dimer monogallates (*m*/*z* 729) were detected in grape canes in previous studies [10,17], although here their positions are proposed for the first time.

Epigallocatechin and gallocatechin (*m*/*z* 305.0665, peak 21; *m*/*z* 305.0662, peak 27) were tentatively identified. Fragmentation of both compounds produced ions at *m*/*z* 261, 221, 219, and 179. The ion at *m*/*z* 261 was due to loss of CO_2_ (−44 Da). The ions at *m*/*z* 221, 219, and 179 arose from cleavage of the A ring and loss of −126 Da by HRF [53]. To the best of our knowledge, this is the first time that epigallocatechin or gallocatechin have been identified in grape canes.

A prodelphinidin dimer formed with units of (epi)catechin and (epi)gallocatechin (EGC) gallate (*m*/*z* 745.1400, peak **35**) was tentatively assigned as (epi)catechin→(epi)gallocatechin gallate (EGCG). MS^2^ of *m*/*z* 745 produced ions at *m*/*z* 593, 575, 457, 441, 423, and 305. The ion at *m*/*z* 593 was generated by the loss of a galloyl moiety (−152 Da), and at *m*/*z* 575 by the loss of gallic acid (−170 Da). The ion at *m*/*z* 457 resulted from QM cleavage and suggested a linkage between (epi)catechin and (epi)gallocatechin gallate [54]. Furthermore, the ion at *m*/*z* 305 generated by QM fission suggests that (epi)catechin and (epi)gallocatechin are positioned at the top and bottom, respectively [55]. The ion at *m*/*z* 593 underwent further fragmentation, producing an ion at *m*/*z* 441 due to RDA (−152 Da) cleavage [55]. The high-intensity ion at *m*/*z* 423 arose from the loss of a water molecule (−18 Da) from the ion at *m*/*z* 441. As far as we know, this is the first time that (epi)catechin—(epi)gallocatechin gallate has been identified in grape canes.

(Epi)gallocatechin gallate (EGCG) (*m*/*z* 457.0770, peak 38) was also tentatively identified. MS^2^ of *m*/*z* 457 produced ions at *m*/*z* 331, 305, and 169. The ions at *m*/*z* 305 and 169 were formed by (epi)gallocatechin and gallic acid deprotonated units, respectively [53]. The ion at *m*/*z* 331 was generated by the HRF (−126 Da) mechanism, characteristic of flavan 3-ol monomers [56]. (Epi)gallocatechin gallate is the predominant polyphenol in green tea, and is largely responsible for the biological activity of this beverage [57]. Widely studied for its antioxidant [58], anticarcinogenic [59], and neuroprotective properties [60], (epi)gallocatechin gallate has been identified and quantified in grape seeds, including of the Pinot Noir variety [61], although to our knowledge, this has not been previously reported in grape canes.

Theaflavin (*m*/*z* 563.1191, peak 47) was likewise tentatively identified. MS^2^ of *m*/*z* 563 produced ions at *m*/*z* 545, 519, 425, 407, 397, and 379. The ions at *m*/*z* 545 and 519 arose from a loss of H_2_O (−18 Da) and CO_2_ (−44 Da), respectively [53]. The ion at *m*/*z* 425 was due to an RDA rearrangement of the *m*/*z* 563 precursor ion with the loss of a neutral molecule (−138 Da). Fragmentation of the ion at *m*/*z* 425 led to ions at *m*/*z* 407, 397, and 379, corresponding to losses of H_2_O (−18 Da), CO (−28 Da), and H_2_O and CO (−46 Da), respectively [53]. Theaflavins can be produced from green tea catechins (EC, ECG, EGC, and EGCG) through oxidation by polyphenol oxidase and peroxidase enzymes. This process occurs in fresh green tea leaves during the production of black tea leaves or the green tea fermentation stage [62]. Accordingly, the presence of theaflavin in grape canes was tentatively attributed to the extraction process, which provoke its formation from other flavan-3-ols. To the best of our knowledge, this is the first report of theaflavin in grape canes.

Four prodelphinidin dimers consisting of (epi)gallocatechin—(epi)catechin (*m*/*z* 593.1305, peak 20; *m*/*z* 593.1301, peak 22; *m*/*z* 593.1296, peak 24; *m*/*z* 593.1290, peak 34) were also tentatively identified. Three (epi)gallocatechin→(epi)catechins were observed at peaks 20, 24, and 34. The MS^2^ spectrum of this sequence produced ions at *m*/*z* 467, 425, 407, 303, and 289. The ion at *m*/*z* 467 was due to fragmentation by HRF [M − H − 126]^−^ on the upper unit. The ion at *m*/*z* 425 arose from RDA cleavage on the extension unit of the dimer [63], and at *m*/*z* 407 from water loss at *m*/*z* 425. These dimers were identified as (epi)gallocatechin→(epi)catechin based on the specific ions at *m*/*z* 303 and 289 derived from QM cleavage [52,63]. Peak 22 of the (epi)catechin→(epi)gallocatechin sequence showed distinctive ions at *m*/*z* 441, 423, 305, and 287. The ions at *m*/*z* 441 and 423 were generated by the RDA mechanism and the subsequent loss of a water molecule [64]. The ions at *m*/*z* 305 and 287 resulted from QM cleavage and were specific to the (epi)catechin→(epi)gallocatechin sequence [52]. Prodelphinidin dimers (*m*/*z* 593) have been detected in grape cane extracts in previous studies [10,17], although their sequences are proposed here for the first time.

Two prodelphinidin dimers consisting of (epi)gallocatechin→(epi)gallocatechin (*m*/*z* 609.1244, peak **17**; *m*/*z* 609.1240, peak **18**) were tentatively identified. The MS^2^ spectrum of *m*/*z* 609 produced ions at *m*/*z* 483, 441, 423, and 305. The ion at *m*/*z* 483 was due to HRF [M − H − 126]^−^ on the upper unit. The ion at *m*/*z* 441 can be attributed to RDA-type fragmentation and at *m*/*z* 423 to water elimination from *m*/*z* 441. The ion at *m*/*z* 305 was produced by QM cleavage between the C and D rings [64]. Prodelphinidin dimers made up of two (epi)gallocatechin units have been previously identified in red wine [52,64], although not in grape canes.

**Figure 4:**
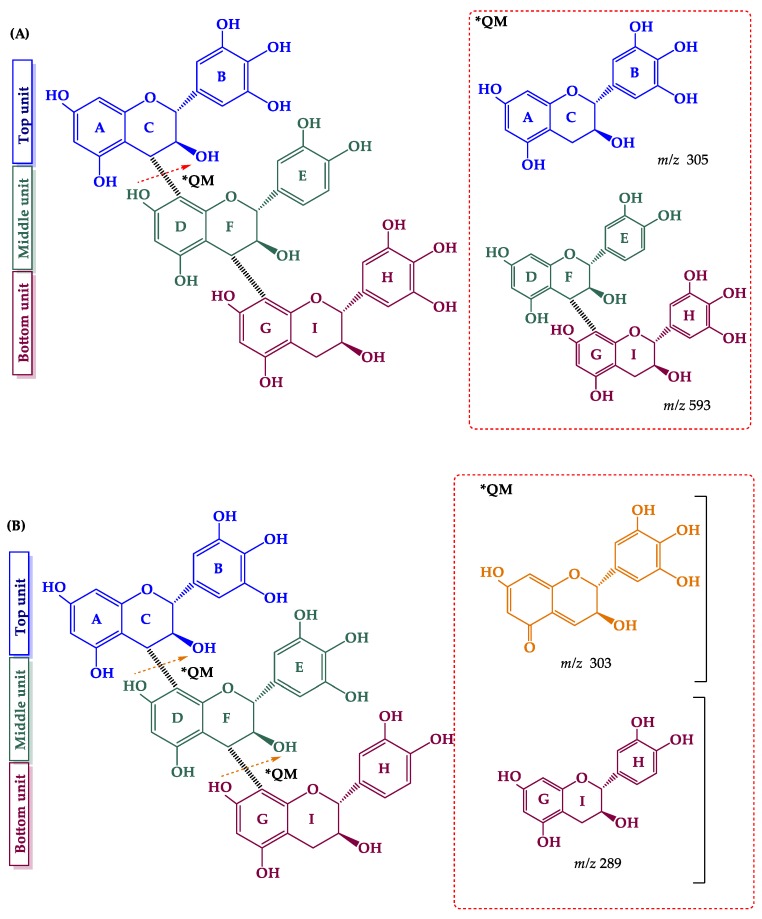
Fragmentation pathways of (epi)gallocatechin→(epi)catechine→(epi)gallocatechin (**A**) and (epi)gallocatechin→(epi)gallocatechin→(epi)catechin (**B**): key ions produced by quinone methide (QM) fragmentation.

Two prodelphinidin trimers (*m*/*z* 897.1869, peak 23; *m*/*z* 897.1868, peak 25) were tentatively identified. A trimer with the sequence (epi)gallocatechin→(epi)catechin→(epi)gallocatechin was detected at peak 23, with ions at *m*/*z* 771, 729, 711, 593, and 305. The ion at *m*/*z* 771 was generated by HRF [M − H − 126]^−^ of the C ring. The ion at *m*/*z* 729 was produced by an RDA-type mechanism on the upper unit, and the consequent loss of a water molecule led to *m*/*z* 711. The ions at *m*/*z* 593 and 305 were due to QM cleavage between the C and D rings [64] (Figure 4A), and were specific to the proposed sequence. Peak 25 corresponds to (epi)gallocatechin→(epi)gallocatechin→(epi)catechin, with ions at *m*/*z* 771, 729, 711, 593, 303, and 289. Specific ions at *m*/*z* 303 and 289 were detected, thereby indicating a QM cleavage of the interflavan bonds and formation of monomeric units, (epi)gallocatechin (-3H) and (epi)catechin, respectively (Figure 4B) [65]. A prodelphinidin trimer (*m*/*z* 897) was previously identified in a grape cane extract [10]. The sequences of the two prodelphinidin trimers identified here are proposed for the first time.

### 2.4. Flavonols and Derivatives

Flavonols are biologically valuable phytochemicals associated with antioxidant and anticancer activities. In particular, myricetin has shown a wide spectrum of biological properties, including antioxidant, anticancer, anti-inflammatory, and possibly even protection against Parkinson’s and Alzheimer’s disease [66]. Quercetin, on the other hand, has attracted attention for its potential effects against cardiovascular diseases [67]. Three conjugated flavonols were identified in the chromatograms of grape cane extracts.

Myricetin-*O*-hexoside (*m*/*z* 479.0821, peak 48) showed ions at *m*/*z* 317 and 316 corresponding to the loss of a hexoside moiety (−162 Da) with concomitant H rearrangement, as usually occurs with polyphenol *O*-glycosides [68]. Myricetin-*O*-hexoside was tentatively identified by comparison with the mass spectra of previous studies using the LTQ-Orbitrap to analyze red wine [25], persimmon leaves [28], and grape pomace [27]. Although myricetin has been previously identified and quantified in vine shoot extracts [39], to our knowledge, this is the first identification of its hexoside derivatives in grape canes.

Quercetin-*O*-glucoside (*m*/*z* 463.0876, peak 49) was unambiguously determined and confirmed by comparison with its pure standard. The MS^2^ spectrum of *m*/*z* 463 produced ions at *m*/*z* 301 and 299 due to the loss of a hexoside moiety (−162 Da) and concomitant H rearrangement, respectively. Quercetin-*O*-hexoside was detected previously in grape canes using a QTrap3200 LC/MS/MS system [17].

Quercetin-3-*O*-glucuronide (*m*/*z* 477.0674, peak 50) was also identified. The MS^2^ spectrum of *m*/*z* 477 revealed an ion at *m*/*z* 301 arising from the loss of a glucuronide moiety (−176 Da). Quercetin-3-*O*-glucuronide has been previously identified in grape canes infected by Bois noir, a serious grapevine yellows disease [19].

### 2.5. Flavanonols and Derivatives

Flavanonols, also known as dihydroflavonols, are a polyphenol subclass inversely associated with diabetes in animal and in vitro models [69]. Furthermore, a high intake of dihydroflavonols has been linked with a reduced risk of diabetes in elderly persons at high risk of cardiovascular disease [70]. Thus, flavanonols, particularly dihydroquercetin (or taxifolin), have high potential value for the development of new natural drugs for the control of type 2 diabetes.

**Figure 5:**
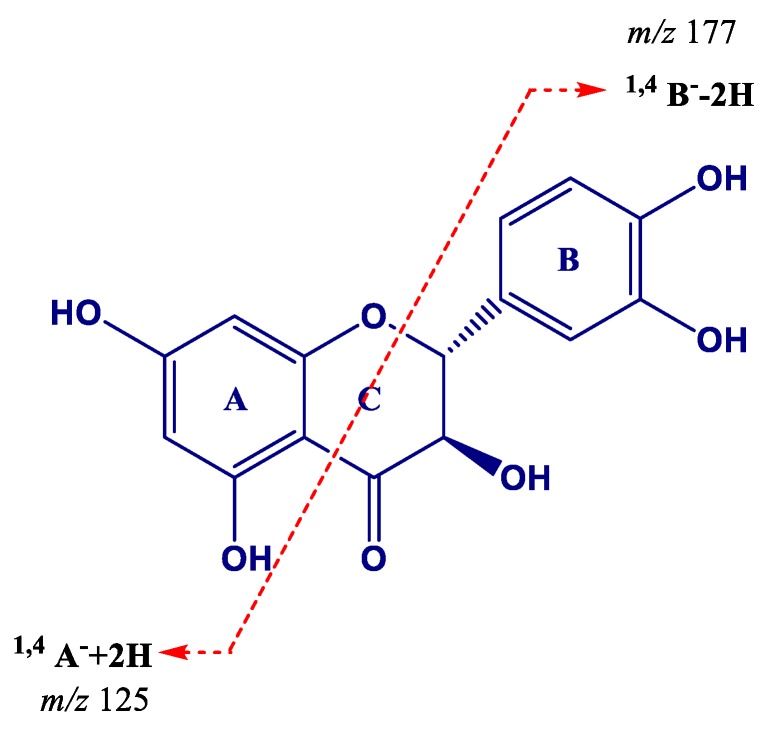
Proposed formation of product ions at *m*/*z* 177 and 125 for taxifolin (dihydroquercetin).

Three flavanonols were identified in the grape cane extract. Taxifolin (*m*/*z* 303.0505, peak **51**) showed ions at *m*/*z* 285, 177, and 125. The ion at *m*/*z* 285 was due to the loss of a water molecule (−18 Da), whereas at *m*/*z* 177 and 125 the ions correspond to cleavage of the C ring attributed to ^1,4^B^−^ − 2H, and ^1,4^A^−^ + 2H scissions, respectively (Figure 5) [71]. This is the first report of taxifolin in extracts from grape canes.

Two isomers of astilbin (*m*/*z* 449.1090, peak 52; *m*/*z* 449.1086, peak 53) were also tentatively identified. The MS^2^ spectrum of *m*/*z* 449 produced ions at *m*/*z* 303, 285, and 151. Those at *m*/*z* 303 and 285 were generated by the loss of a rhamnose moiety (−146 Da) and the consecutive loss of a water molecule (−18 Da), respectively. The ion at *m*/*z* 151 was generated after RDA-type cleavage [72]. Astilbin (dihydroquercetin-rhamnoside) (*m*/*z* 449) has been previously identified in grape canes [19].

### 2.6. Flavanones and Derivatives

Eriodictyol and its glycoside derivatives are the main flavanones found in grape canes. Eriodictyol protects against oxidative stress and could have potential application in nutraceuticals for the prevention of cardiovascular disease [73]. Eriodictyol and two of its glycoside conjugates were identified in the grape cane extract.

The MS^2^ spectrum of eriodictyol-*O*-hexoside (*m*/*z* 449.1090, peak 54; *m*/*z* 449.1087, peak 55) produced an ion at *m*/*z* 287 generated by the loss of a hexosyl moiety (−162 Da). This is the first time that eriodictyol-*O*-hexoside is reported from grape canes.

**Figure 6:**
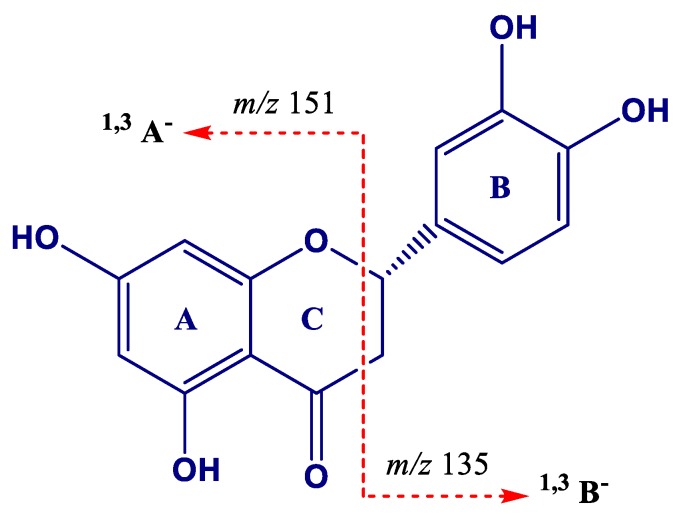
Proposed formation of product ions at *m*/*z* 135 and 151 for eriodictyol.

Eriodictyol (*m*/*z* 287.0556, peak 56) showed product ions at *m*/*z* 151 and 135 formed by an RDA-type fragmentation in the C ring involving type ^1,3^A^−^ and ^1,3^B^−^ scission (Figure 6) [74], respectively. Eriodictyol has been previously identified in elicited *V. vinifera* (Pinot Noir) hairy root culture extracts by LC-MS and ^13^C NMR methods [21].

### 2.7. Stilbenes and Derivatives

Several authors have reported stilbenes as well as oligostilbenoids in grape canes [9,10,17,75]. The most relevant stilbene is resveratrol, associated with activity against cardiovascular diseases, neurodegenerative diseases, and cancer [76]. In grape canes, the most abundant polyphenolic compounds are oligomeric stilbenes (oligostilbenoids) [9].

#### 2.7.1. Stilbene Monomers

Resveratrol (*m*/*z* 227.0707, peak 67) was confirmed by comparison with an available standard. The MS^2^ spectrum of *m*/*z* 227 showed ions at *m*/*z* 185 [M − H − 42]^−^ and 143 [M − H − 42 − 42]^−^ produced by the sequential loss of two ketene molecules (C_2_H_2_O) [77].

Piceatannol (*m*/*z* 243.0659, peak 62) was also identified by comparison with its pure standard. The MS^2^ spectrum of *m*/*z* 243 produced ions at *m*/*z* 225 [M − H − 18]^−^, arising from the loss of a water molecule, and at *m*/*z* 201 [M − H − 42]^−^ and 159 [M − H − 42 − 42]^−^ due to successive losses of C_2_H_2_O. Both monomers has been previously identified in grape cane extract [9,11,17,18].

#### 2.7.2. Stilbene Dimers

Four different resveratrol dimers were identified. (i) Pallidol *(m*/*z* 453.1340, peak 66) showed ions at *m*/*z* 359 [M − H − 94]^−^ and 265 [M − H − 94 − 94]^−^ produced by successive losses of one and two phenol groups, respectively [78]. (ii) (*E*)-ε-viniferin (*m*/*z* 453.1335, peak 73) was identified by comparison with its pure standard. The MS^2^ spectrum of (*E*)-ε-viniferin showed ions at *m*/*z* 359 [M − H − 94]^−^, produced by the loss of a phenol group, and at *m*/*z* 347 [M − H − 106]^−^, by the loss of 4-methylenecyclohexa-2,5-dienone [78]. (iii) (*E*)-ω-viniferin (*m*/*z* 453.1335, peak 74) was tentatively identified and showed ions at *m*/*z* 435 [M − H − 18]^−^ and 411 [M − H − 42]^−^, produced by the loss of a water molecule and a C_2_H_2_O group, respectively, and at *m*/*z* 359 and 347, as explained above. (iv) The stilbenoid dimer (*m*/*z* 453.1339, peak **68**) was tentatively identified as a resveratrol dimer and showed a high intensity ion at *m*/*z* 359 [M − H − 94]^−^ due to the loss of a phenol group. Several stilbenoid dimers with a very similar structure, parthenocissin A, quadrangularin A, and ampelopsin D, are reported in the literature. Therefore, the accurate identity of this compound should be elucidated by NMR spectroscopy techniques [33,79]. Resveratrol dimers have been previously identified in grape cane extracts [9,17].

Three stilbenoid heterodimers consisting of (*E*)-resveratrol and (*E*)-piceatannol with signals at *m*/*z* 469 were also detected. Stilbenoid dimer 1 (*m*/*z* 469.1283, peak **61**) revealed ions at *m*/*z* 451 [M − H − 18]^−^ and 375 [M − H − 94]^−^, due to the loss of a water molecule and a phenol moiety, respectively, and at *m*/*z* 363 [M − H − 106]^−^ due to the loss of C_7_H_6_O. Stilbenoid dimer 2 (*m*/*z* 469.1287, peak **64**) was also tentatively identified and showed ions at *m*/*z* 363 and 375, explained above. Stilbenoid dimer 3 (*m*/*z* 469.1283, peak **71**) revealed ions at *m*/*z* 385 [M − H − 84]^−^ (the loss of two C_2_H_2_O moieties) [80], at *m*/*z* 359 [M − H − 110]^−^ (the loss of the pyrocatechol or resorcinol moiety) [9,80], and at *m*/*z* 347 [M − H − 122]^−^ (the loss of C_7_H_6_O_2_). The latter ion was observed by Moss et al. [78] and Sáez et al. [9] for scirpusin A.

Four oxidized dimers with signals at *m*/*z* 471 were tentatively identified. Restrisol (A or B) (*m*/*z* 471.1441, peak **58**) revealed ions at *m*/*z* 377 [M − H − 94]^−^, due to the loss of a phenolic group, at *m*/*z* 349 [M − H − 94 − 28]^−^, produced by the consecutive loss of a phenol group (−94 Da) and carbon monoxide CO (−28 Da), and at *m*/*z* 255 [M − H − 94 − 28 − 94]^−^, due to the successive loss of phenol (−94 Da), CO (−28 Da) and phenol (−94 Da) groups [78]. Another three compounds with the same signals at *m*/*z* 471 were assigned as oxidized dimers (1 to 3) (*m*/*z* 471.1438, peak **59**; *m*/*z* 471.1443, peak **60**; *m*/*z* 471.1446, peak **63**), and revealed a prominent ion at *m*/*z* 349 analogous to restrisol. On the other hand, restrisol and oxidized stilbenoid dimers could be formed by dimerization of resveratrol induced by the laccase enzymes or its isoform produced by the mycopathogen *Botrytis cinerea* [81]. Oxidized dimers have also been previously identified in grape canes [9,17].

#### 2.7.3. Glycosylated Stilbenes

Resveratrol *C*-hexoside (*m*/*z* 389.1241, peak 57) was tentatively identified. The MS^2^ spectrum of *m*/*z* 389 showed ions at *m*/*z* 269 and 299 with losses of 120 Da and 90 Da, respectively, which is the typical fragmentation pattern for C-glycosides in MS^2^ mode [82]. Furthermore, another ion at *m*/*z* 241 was observed with the same relative abundance as reported by Püssa et al. [83]. Resveratrol *C*-hexoside designated as *E*-3,5,4′-trihydroxystilbene 2-*C*-glucoside has been previously identified in grape canes [75].

Resveratrol dimer-*O*-hexoside (*m*/*z* 615.1866, peak 69) was also tentatively identified. MS^2^ of *m*/*z* 615 showed a high intensity ion at *m*/*z* 453 [M − H − 162]^−^ produced by the loss of a hexoside moiety. To our knowledge, this is the first resveratrol dimer-*O*-hexoside identified in grape cane extract.

Viniferin diglycoside (*m*/*z* 777.2387, peak 65) was also tentatively identified. The MS^2^ of *m*/*z* 777 showed ions at *m*/*z* 615 [M − H − 162]^−^ and 453 [M − H − 162 − 162]^−^ corresponding to the loss of one hexoside unit and two hexoside units, respectively. Viniferin diglycoside has been previously identified in grape canes [10].

#### 2.7.4. Stilbene Oligomers

Hopeaphenol and isohopeaphenol (*m*/*z* 905.2582, peak 70; *m*/*z* 905.2580, peak 72) were confirmed after comparison with the previously isolated standards [9]. The two compounds showed the same ions at *m*/*z* 811 and 717, and their identification was confirmed based on the elution time order. The ions at *m*/*z* 811 [M − H − 94]^−^ and 717 [M − H − 94 − 94]^−^ were produced by the loss of one and two phenol moieties, respectively [78].

A stilbenoid tetramer (*m*/*z* 905.2576, peak 75), tentatively identified, revealed ions at *m*/*z* 887, 811, 799, and 359. The ion at *m*/*z* 887 [M − H − 18]^−^ was produced by the loss of a water molecule and at *m*/*z* 811 [M − H − 94]^−^ by the loss of a phenolic group. The ion at *m*/*z* 799 was probably due to the loss of 4-methylenecyclohexan-2,5-dienone (−106 Da), as proposed by Moss et al. [78]. The ion at *m*/*z* 359 was due to the loss of a phenolic group from a previously divided tetramer molecule [78]. Stilbenoid tetramers has been previously identified in grape canes [9,10,17].

## 3. Materials and Methods

### 3.1. Chemicals

Acetonitrile, formic acid, water, and ethanol were purchased from Merck (Darmstadt, Germany). All solvents were of HPLC grade. Ultrapure water was obtained from a Milli-Q water purification system (Millipore, Bedford, MA, USA).

Gallic, ellagic, and 4-hydroxybenzoic acid, (+)-catechin, (−)-epicatechin, (*E*)-resveratrol, (*E*)-piceatannol, quercetin-*O*-glucoside, and (*E*)-ε-viniferin were purchased from Sigma-Aldrich (St. Louis, MO, USA). Epicatechin gallate was acquired from Extrasynthèse (Genay, France). All standards were handled without exposure to light.

### 3.2. Grape Cane Samples

Grape canes (*Vitis vinifera* L.) of the variety Pinot Noir were collected from healthy plants in an organic vineyard (chemical fertilizers, pesticides, fungicides, not employed), Viña De Neira, located in Ránquil, Itata Valley, the Biobio region in South Chile (36°36′50.33″ S, 72°39′40.63″ W at 279 m of altitude). After pruning, all samples were cut in 30–50 cm pieces and stored at room temperature (~20 °C) for at least three months [9,10].

### 3.3. Polyphenol Extraction from Grape Canes

Grape canes were handled in a room with light filters to prevent photodegradation and oxidation of the polyphenols. The extraction was done following a previously reported procedure with minor modifications [82].

Grape canes (0.5 g, *n* = 3) were homogenized and vortexed for 1 min with 4 mL ethanol/water (80:20, *v*/*v*) and then sonicated in an ultrasound bath (Bandelin electronic GmbH&Co.KG, Berlin, Germany) for 10 min. The grape cane extract was centrifuged at 4000 RPM for 5 min at 4 °C. The supernatant was collected and the extraction procedure was repeated twice. The supernatants were combined and evaporated under nitrogen flow, and the residue was reconstituted into 0.1% of aqueous formic acid (5 mL). The extract was filtered by 0.20 µm PTFE (Waters Corporation, Mildfore, MA, USA) into an amber vial. Samples were stored at −20 °C until analysis by LC LTQ-Orbitrap.

### 3.4. LC-LTQ-Orbitrap-MS Analyses

Liquid chromatography analysis was performed using an Accela chromatograph (Thermo Scientific, Hemel Hempstead, UK) equipped with a quaternary pump, a photodiode array detector (PDA), and a thermostated autosampler. Chromatographic separation was performed in an Atlantis T3 column 2.1 × 100 mm, 3µm (Waters, Milford, MA, USA). Gradient elution of analytes was carried out with H_2_O/0.1% H-COOH (solvent A) and CH_3_CN (solvent B) at a constant flow rate of 0.350 mL/min, and the injection volume was 5 µL. A non-linear gradient was used: 0 min, 2% B; 0–2 min, 8% B; 2–12 min, 20% B; 12–13 min, 30% B; 13–14 min, 100% B; 14–17 min, 100% B; 17–18 min, 2% B and the column was equilibrated for 5 min to initial conditions [28].

The LC system was coupled to an LTQ-Orbitrap Velos mass spectrometer (Thermo Scientific, Hemel Hempstead, UK) used for accurate mass measurements and equipped with an ESI source operated in negative mode. Operation parameters were as follows: source voltage, 4 kV; sheath gas, 20 a.u. (arbitrary units); auxiliary gas, 10 a.u.; sweep gas, 2 a.u.; and capillary temperature, 275 °C. Default values were used for most other acquisition parameters (FT Automatic gain control (AGC) target 5 × 10^5^ for MS mode and 5·× 10^4^ for MS^n^ mode). Grape cane samples were analyzed in full scan mode at a resolving power of 30,000 (FWHM at *m*/*z* 400) and data-dependent MS/MS events acquired at a resolving power of 15,000. The most intense ions detected in the full scan spectrum were selected for the data-dependent scan. Parent ions were fragmented by high-energy C-trap dissociation (HCD) with normalized collision energy of 35 V and an activation time of 10 ms. The mass range in Fourier transformation mass spectrometry (FTMS) mode was from *m*/*z* 100 to 1000 [28]. Instrument control and data acquisition were performed with Xcalibur 3.0 software (Thermo Fisher Scientific).

## 4. Conclusions

The use of LC-LTQ-Orbitrap-MS allowed a comprehensive profiling of polyphenols in a grape cane extract. The characterization was carried out based on accurate mass measurement with low error (<3.1 ppm) and MS^2^ spectrum data. The polyphenolic compounds were confirmed by comparisons with pure standards whenever possible, as well as by referring to the literature. A total of 75 polyphenolic compounds were identified or tentatively characterized, 17 of them reported for the first time in grape canes. Most of the identified polyphenols were hexoside derivatives, such as syringic acid hexoside, hydroxybenzoyl hexoside, ellagic acid hexoside, myricetin-*O*-hexoside, eriodictyol-*O*-hexoside, and resveratrol dimer-*O*-hexoside. Additionally, an exhaustive analysis of proanthocyanidins showed for the first time the presence of pentameric procyanidins and (epi)gallocatechins, and the specific sequence of each prodelphinidin compound.

The reported results broaden knowledge of the polyphenol profile of grape canes and may be useful for further investigations related to the production of high-added-value food additives based on this by-product.

## Figures and Tables

**Table 1:** Phenolic compounds identified in a grape cane extract by liquid chromatography coupled with electrospray ionization hybrid linear trap quadrupole-Orbitrap mass spectrometry (LC-ESI-LTQ-Orbitrap-MS) in negative mode.

Peak	Compounds	R.T. (min)	Accurate Mass [M − H]^−^	MS/MS Ions (% Intensity)	Δm (ppm)	Ion Molecular Formula (IMF)
**Hydroxybenzoic Acids and Derivatives**						
1	Monogalloyl-glucose (**1**)	3.60	331.0668	271.0446(60), 211.0237(20), 169.0133(100)	−0.936	C_13_H_15_O_10_
2	Gallic acid *	4.30	169.0141	125.0239(100)	−0.985	C_7_H_5_O_5_
3	Monogalloyl-glucose (**2**)	5.38	331.0664	271.0446(60), 211.0237(20), 169.0133(100)	−1.510	C_13_H_15_O_10_
4	Protocatechuic acid-*O*-hexoside (**1**)	7.39	315.0719	153.0185(100)	−1.001	C_13_H_15_O_9_
5	Protocatechuic acid	7.66	153.0192	109.0290(100)	−1.058	C_7_H_5_O_4_
6	Protocatechuic acid-*O*-hexoside (**2**)	8.52	315.0718	153.0186(100)	−0.969	C_13_H_15_O_9_
7	Syringic acid hexoside	8.75	359.0981	197.0446(100)	−0.724	C_15_H_19_O_10_
8	2-Hydroxybenzoic acid	9.47	137.0243	93.0341(100)	−1.075	C_7_H_5_O_3_
9	Hydroxybenzoyl hexoside	9.96	299.0770	137.0236(100)	−1.039	C_13_H_15_O_8_
10	4-Hydroxybenzoic acid *	10.02	137.0242	93.0340(100)	−1.221	C_7_H_5_O_3_
11	Ellagic acid hexoside	13.99	463.0518	300.9974(100)	0.079	C_20_H_15_O_13_
12	Gallic acid ethyl ester	14.28	197.0454	169.0135(100)	−1.252	C_9_H_9_O_5_
13	Ellagic acid pentoside	16.03	433.0416	300.9975(100), 299.9898(40)	−0.766	C_19_H_13_O_12_
14	Ellagic acid *	16.94	300.9980	257.0079(100), 229.0132(60), 185.0235(30)	−1.131	C_14_H_5_O_8_
**Hydroxycinnamic Acids Derivatives**						
15	Caftaric acid	9.27	311.0406	179.0343(45), 149.0965(100)	−0.852	C_13_H_11_O_9_
16	Coutaric acid	11.18	295.0456	163.0392(100)	−1.052	C_13_H_11_O_8_
**Flavanols (Proanthocyanidins)**						
17	(Epi)gallocatechin (EGC)→(epi)gallocatechin (**1**)	5.69	609.1244	483.0917(20), 441.0812(85), 423.0707(100), 305.0656(45)	−0.966	C_30_H_25_O_14_
18	(Epi)gallocatechin→(epi)gallocatechin (**2**)	7.81	609.1240	483.0913(25), 441.0808(100), 423.0703(90), 305.0653(40)	−1.574	C_30_H_25_O_14_
19	Procyanidin trimer (**1**)	7.90	865.1982	739.1638(15), 713.1479(20), 695.1379(100), 577.1329(25), 451.1016(15), 425.0867(15), 407.0761(20), 289.0709(10)	−1.118	C_45_H_37_O_18_
20	(Epi)gallocatechin→(epi)catechin (**1**)	8.01	593.1305	467.0970(30), 425.0868(100), 407.0763(80), 303.0501(10), 289.0710(70)	0.718	C_30_H_25_O_13_
21	(Epi)gallocatechin (**1**)	8.30	305.0665	261.0761(45), 221.0448(75), 219.0658(65), 179.0344(100)	0.303	C_15_H_13_O_7_
22	(Epi)catechin→(epi)gallocatechin	8.71	593.1301	467.0973(55), 441.0817(40), 423.0715(100), 305.0660(60), 287.0554(10)	0.010	C_30_H_25_O_13_
23	(Epi)gallocatechin→(epi)catechin→(epi)gallocatechin	9.09	897.1869	771.1526(25), 729.1426(15), 711.1321(100), 593.1277(30), 305.0651(5)	−1.445	C_45_H_37_O_20_
24	(Epi)gallocatechin→(epi)catechin (**2**)	9.73	593.1296	467.0970(25), 425.0868(100), 407.0761(50), 303.0510(5), 289.0709(40)	−0.715	C_30_H_25_O_13_
25	(Epi)gallocatechin→(epi)gallocatechin→(epi)catechin	9.86	897.1868	771.1538(15), 729.1435(25), 711.1331(100), 593.1280(20), 303.0499(10), 289.0706(10)	−1.779	C_45_H_37_O_20_
26	Procyanidin dimer (**1**)	10.44	577.1342	451.1026 (95), 425.0872 (80), 407.0766 (95), 289.0711(100)	−1.610	C_30_H_25_O_12_
27	(Epi)gallocatechin (**2**)	10.73	305.0662	261.0762(40), 221.0449(75), 219.0659(60), 179.0345(100)	1.122	C_15_H_13_O_7_
28	Procyanidin dimer (**2**)	10.89	577.1347	451.1027(75), 425.0873(100), 407.0768(90), 289.0713(70)	−0.657	C_30_H_25_O_12_
29	Catechin *	11.39	289.0715	245.0810(100), 205.0498(40), 179.1342(20)	−0.800	C_15_H_13_O_6_
30	Procyanidin trimer (**2**)	11.95	865.1978	739.1652(40), 713.1495 (30), 695.1393(100), 577.1340(50), 451.1024(30), 425.0870(25), 407.0764(55), 289.0710 (20)	−0.841	C_45_H_37_O_18_
31	Procyanidin tetramer	12.13	576.1271 [M − 2H]^2−^	1027.2257(35), 865.1948(30), 863.1793(65), 739.1640(30), 451.1016(45), 407.0756(25), 289.0705(100), 287.0548(40)	−0.424	C_60_H_50_O_24_
32	Procyanidin dimer (**3**)	12.28	577.1348	451.1020(50), 425.0867(100), 407.0761(90), 289.0708(45)	−0.883	C_30_H_25_O_12_
33	Procyanidin dimer (**4**)	12.57	577.1356	451.1026 (65), 425.0872(100), 407.0766(95), 289.0711(60)	−0.815	C_30_H_25_O_12_
34	(Epi)gallocatechin→(epi)catechin (**3**)	12.77	593.1290	467.0968(30), 425.0865(100), 407.0758(70), 303.0500(10), 289.0707(40)	−1.713	C_30_H_25_O_13_
35	(Epi)catechin→(epi)gallocatechin gallate (EGCG)	12.81	745.1400	593.1257(80), 575.1136(55), 457.0757(25), 441.0809(5), 423.0703(100), 305.0655(15)	−2.003	C_37_H_29_O_17_
36	Procyanidin pentamer (**1**)	13.04	720.1580 [M − 2H]^2−^	1315.2897(25), 1153.2595 (25), 1151.2442(55), 1027.2273(25), 865.1955(60), 863.1794(100), 739.1645(25), 635.6298(80), 577.1333(90), 575.1178(80), 451.5990(30), 407.0758(45), 289.0707(70), 287.0550 (40)	0.504	C_75_H_62_O_30_
37	Epicatechin *	13.36	289.0715	245.0810(100), 205.0498(40), 179.1342(10)	−0.766	C_15_H_13_O_6_
38	(Epi)gallocatechin gallate (EGCG)	13.74	457.0770	331.0445(70), 305.0653(35), 169.0135(100)	−1.454	C_22_H_17_O_11_
39	(Epi)catechin gallate (ECG)→(epi)catechin (**1**)	13.86	729.1459	603.1140(5), 577.1345(100), 439.066(5), 425.0876(25), 407.0769(50), 289.0713(10)	−0.230	C_37_H_29_O_16_
40	Procyanidin dimer (**5**)	14.01	577.1346	451.1025(55), 425.0872(90), 407.0766(100), 289.0711(50)	−0.883	C_30_H_25_O_12_
41	Procyanidin trimer (**3**)	14.39	865.1979	739.1643(40), 713.1486(35), 695.1385(100), 577.1334(55), 451.1020(30), 425.0866(25), 407.0760(50), 289.0708(15)	−0.760	C_45_H_37_O_18_
42	(Epi)catechin→(epi)catechin gallate	14.84	729.1449	603.1133(30), 577.1312(35), 559.0917(30), 451.1024(45), 441.0818(30), 407.0764(100), 289.0710(30)	−1.629	C_37_H_29_O_16_
43	Procyanidin trimer (**4**)	15.43	865.1959	739.1643(50), 713.1489(20), 695.1384(100), 577.1334(50), 451.1020(25), 425.0866(25), 407.0759(55), 289.0707(10)	−3.094	C_45_H_37_O_18_
44	Procyanidin pentamer (**2**)	15.59	720.1578 [M − 2H]^2−^	1315.2906(25), 1153.2585(20), 1151.2437(45), 1027.2261(20), 865.1949(50), 863.1793(75), 739.1639(20), 635.6275(100), 577.1330(60), 575.1175(60), 451.1016(20), 407.0756(25), 289.0706(45), 287.0548(25)	−0.079	C_75_H_62_O_30_
45	Epicatechin gallate *	16.89	441.0825	289.0710(100), 169.0137(30)	−0.476	C_27_H_17_O_10_
46	(Epi)catechin gallate→(epi)catechin (**2**)	17.54	729.1441	603.1125(5), 577.1332(100), 439.0657(5), 425.0866(25), 407.0760(30), 289.0705(5)	2.184	C_37_H_29_O_16_
47	Theaflavin	20.88	563.1191	545.1064(100), 519.1272(45), 425.0857(40), 407.0751(65), 397.0908(30), 379.0805(60)	−1.295	C_29_H_23_O_12_
**Flavonols**						
48	Myricetin-*O*-hexoside	15.49	479.0821	317.0288(60), 316.0211(100)	−1.239	C_21_H_19_O_13_
49	Quercetin-*O*-glucoside *	17.23	463.0876	301.0337(100), 299.0174(30)	−1.402	C_21_H_19_O_12_
50	Quercetin-3-*O*-glucuronide	17.28	477.0674	301.0341(100)	−0.700	C_21_H_17_O_13_
**Flavanonol**						
51	Taxifolin	17.01	303.0505	285.0391(100), 177.0185(10), 125.0239(10)	−1.636	C_15_H_11_O_7_
52	Astilbin (**1**)	17.39	449.1090	303.0497(100), 285.0391(90), 151.0030(30)	−0.211	C_21_H_21_O_11_
53	Astilbin (**2**)	18.22	449.1086	303.0497(100), 285.0391(85), 151.0030(25)	−0.612	C_21_H_21_O_11_
**Flavanones**						
54	Eriodictyol-*O*-hexoside (**1**)	13.58	449.1090	287.0545(100)	−0.367	C_21_H_21_O_11_
55	Eriodictyol-*O*-hexoside (**2**)	19.03	449.1087	287.0548(100)	−0.433	C_21_H_21_O_11_
56	Eriodictyol	21.08	287.0556	151.0033(100), 135.0448(10)	−1.677	C_15_H_11_O_6_
**Stilbenoids**						
57	Resveratrol *C*-hexoside	13.66	389.1241	269.0812(100), 241.0864(10), 299.0915(5)	−0.208	C_20_H_21_O_8_
58	Restrisol (A or B)	15.15	471.1441	377.1015(90), 349.1067(100), 255.0651(80)	−1.754	C_28_H_23_O_7_
59	Oxidized stilbenoid dimer (**1**)	16.25	471.1438	349.1066(100)	−2.391	C_28_H_23_O_7_
60	Oxidized stilbenoid dimer (**2**)	16.85	471.1443	349.1066(100)	−1.351	C_28_H_23_O_7_
61	Stilbenoid dimer (**1**)(heterodimer)	17.78	469.1283	451.1181(100), 375.0866(30), 363.0869(35)	−1.484	C_28_H_21_O_7_
62	(*E*)-Piceatannol *	18.01	243.0659	225.0551(100), 201.0551(65), 159.0447(20)	−1.202	C_14_H_11_O_4_
63	Oxidized stilbenoid dimer (**3**)	18.93	471.1446	349.1067(100)	−2.709	C_28_H_23_O_7_
64	Stilbenoid dimer (**2**)(heterodimer)	19.23	469.1287	375.0855(20), 363.0857(100)	−1.122	C_28_H_21_O_7_
65	Viniferin diglycoside	19.33	777.2387	615.1848(95), 453.1327(100)	−1.670	C_40_H_41_O_16_
66	Pallidol	19.51	453.1340	359.0915(100), 265.0497(10)	−0.820	C_28_H_21_O_5_
67	(*E*)-resveratrol *	20.27	227.0707	185.0602(65), 143.0496(20)	−2.543	C_14_H_11_O_3_
68	Stilbene dimer (resveratrol+resveratrol)	20.62	453.1339	359.0912(100)	-1.593	C_28_H_21_O_6_
69	Resveratrol dimer-*O*-hexoside	20.85	615.1866	453.1325(100)	−0.951	C_34_H_31_O_11_
70	Stilbenoid tetramer (Hopeaphenol *)	21.17	905.2582	811.2159(100), 717.1748(80)	−2.331	C_56_H_41_O_12_
71	Stilbenoid dimer (3)(Scirpusin A)	21.35	469.1283	451.1182(25), 385.1066(50), 375.0860(100), 359.0912(30), 347.0919(15)	−2.188	C_28_H_21_O_7_
72	Stilbenoid tetramer (Isohopeaphenol *)	21.41	905.2580	811.2158(100), 717.1747(80)	−2.596	C_56_H_41_O_12_
73	(*E*)-ε-viniferin *	21.68	453.1335	359.0924(100), 347.0919(40)	−1.813	C_28_H_21_O_6_
74	(*E*)-ω-viniferin	21.87	453.1335	435.1230(35), 411.1229(25), 359.0918(100), 347.0918(55)	−1.593	C_28_H_21_O_6_
75	Stilbenoid tetramer	22.05	905.2576	887.2472(25), 811.2159(50), 799.2164(100), 359.0917(30)	−3.004	C_56_H_41_O_12_

* Compounds identified by comparison with pure standards; R.T., retention times; Δm, mass measurement error; compounds **31**, **36**, and **44** appeared as doubly-charged ions; isomers are displayed in the bracket.

## Abbreviations

FTMS	fourier transformation mass spectrometry
PDA	photodiode array detector
AGC	automatic gain control
PTFE	polytetrafluoroethylene
HCD	high-energy C-trap dissociation
FWHM	full width at half maximum
a.u.	arbitrary units
ESI	electrospray ionization
HPLC	high performance liquid chromatography
MS	mass spectrometry
LC	liquid chromatography
^13^C-NMR	Carbon-13 nuclear magnetic resonance
EC	catechin
ECG	catechin gallate
EGC	gallocatechin
EGCG	gallocatechin gallate
QTrap	quadrupole ion trap
DP	degree of polymerization
QM	quinone methide
HRF	heterolytic ring fission
RDA	retro-Diels-Alder
IMF	Ion molecular formula
LC–LTQ-Orbitrap	liquid chromatography coupled with electrospray ionization hybrid linear trap quadrupole-Orbitrap mass spectrometry
